# An Artificial Neural Network for Image Classification Inspired by the Aversive Olfactory Learning Neural Circuit in *Caenorhabditis elegans*

**DOI:** 10.1002/advs.202410637

**Published:** 2024-12-16

**Authors:** Xuebin Wang, Chunxiuzi Liu, Meng Zhao, Ke Zhang, Zengru Di, He Liu

**Affiliations:** ^1^ Department of Systems Science Faculty of Arts and Sciences Beijing Normal University Zhuhai 519087 China; ^2^ International Academic Center of Complex Systems Beijing Normal University Zhuhai 519087 China; ^3^ School of Systems Science Beijing Normal University Beijing 100875 China; ^4^ School of Computer Science and Engineering Tianjin University of Technology Tianjin 300384 China

**Keywords:** artificial neural network, *Caenorhabditis elegans*, image classification, neural circuit

## Abstract

This study introduces an artificial neural network (ANN) for image classification task, inspired by the aversive olfactory learning neural circuit in *Caenorhabditis elegans* (*C. elegans*). Although artificial neural networks (ANNs) have demonstrated remarkable performance in various tasks, they still encounter challenges including excessive parameterization, high training costs and limited generalization capabilities, etc. *C. elegans*, boasting a simple nervous system consisting of merely 302 neurons, is capable of exhibiting complex behaviors such as aversive olfactory learning. This research pinpoints key neural circuit related to aversive olfactory learning in *C. elegans* by means of behavioral experiment and high‐throughput RNA sequencing, and then translates it into an architecture of ANN for image classification. Furthermore, other ANNs for image classification with different architectures are constructed for comparative performance analysis to underscore the advantages of the bio‐inspired designed architecture. The results show that the ANN inspired by the aversive olfactory learning neural circuit in *C. elegans* attains higher accuracy, greater consistency and faster convergence rate in the image classification task, particularly when dealing with more complex classification challenges. This study not only demonstrates the potential of bio‐inspired design in improving the capabilities of ANNs but also offers a novel perspective and methodology for future ANNs design.

## Graphical abstract

A neural circuit for aversive olfactory learning in *Caenorhabditis elegans* is abstracted utilizing data from high‐throughput RNA sequencing and connectome reconstruction. Based on this circuit, the architecture of an Artificial Neural Network is modified and deployed in image classification task, demonstrating increased accuracy, improved consistency and accelerated convergence rates relative to other methods.

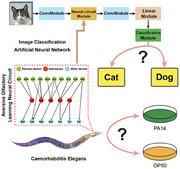

## 1 Introduction

Artificial neural networks (ANNs) are mathematical models inspired by the biological brain, designed to mimic its adaptive learning capabilities. These systems can modify their internal topologies in response to new data, thereby enhancing their performance on a variety of tasks. ANNs have been particularly effective in tackling complex problems such as machine vision,^[^ ^1^, ^2^, ^3^ ^]^ speech recognition^[^ ^4^, ^5^, ^6^ ^]^ and autonomous driving,^[^ ^7^, ^8^ ^]^ which were previously challenging for rule‐based programming methods. Despite their remarkable achievements, there are still several challenges that remain: 1) High‐performance ANNs usually contain an excessive number of parameters, which limits their application scenarios. 2) Training ANNs requires substantial computational resources and time, a process that is growing more costly as the enhancements predicted by Moore's Law diminish.^[^ ^9^ ^]^ 3) The performance of certain ANNs is highly scenario‐specific, exhibiting a significant decline in efficacy when applied to diverse contexts. A crucial point in addressing these challenges is to reduce the parameters, which can bring a series of positive chain reactions to the operation of ANNs. First, reducing the parameters leads to a decreased demand for computing resources. This enables ANNs to show excellent adaptability and operational efficiency whether on powerful server clusters or on edge devices with limited resources. Second, the reduction of parameters can also accelerate the training process of ANNs. Finally, fewer parameters can effectively reduce the risk of overfitting in ANNs.

Currently, the methods for reducing the parameters of ANNs include pruning,^[^ ^10^, ^11^ ^]^ low‐precision quantization,^[^ ^12^, ^13^ ^]^ knowledge distillation^[^ ^14^ ^]^ and model optimization algorithms.^[^ ^15^, ^16^, ^17^, ^18^ ^]^ These methods have achieved positive results. However, they also have their respective deficiencies, such as instability, reliance on experience and lack of interpretability. Brain intelligence‐inspired methods focuses on leveraging principles derived from the circuity mechanisms of the brain to optimize artificial intelligence systems, particularly in reducing the number of parameters required for effective learning and decision‐making. In this study, in light of the sophistication of the biological brain, we draw inspiration from it and create a natural and interpretable method for reducing the parameters of ANNs. The biological brain is very powerful and efficient.^[^ ^19^, ^20^ ^]^ Even the tiniest organisms in the natural world, such as worms, fruit flies and ants, exhibit remarkable capabilities in locomotion and cognition.^[^ ^21^, ^22^ ^]^ *Caenorhabditis elegans* (*C. elegans*) serves as a paradigmatic model in neurobiological research and is notable for its simplicity as it has a nervous system consisting of just 302 neurons. Previous studies have discovered the detailed connectomes of C. elegans neural networks and abstracted several structural features.^[^ ^23^, ^24^ ^]^ Lechner et al have shown that a modified ANN constructed by imitating the nervous system of *C. elegans* can enable the automatic driving of cars with only 19 neurons.^[^ ^25^, ^26^, ^27^ ^]^ These researches merely imitated the computational steps of neural information without referring to the actual neural circuits in *C. elegans*. The neural circuits, which are functional units obtained through long‐term evolutionary screening by *C. elegans*, feature compact structures and high computing speed.

Learning, as an important mechanism for adapting to the environment and enhancing survival ability, is of profound significance for the survival and development of living beings.^[^ ^28^, ^29^ ^]^ Although *C. elegans* possess only a simple nervous system, they can learn to avoid the harm caused by the aversive odors of pseudomonas aeruginosa (PA14).^[^ ^30^, ^31^ ^]^ Learning to avoid aversive odors considerably reveals the working process of the brain of *C. elegans*. First, PA14 odors are detected by sensory neurons which are responsible to these odors. These sensory neurons then transmit the neural information to the downstream interneurons. Subsequently, the interneurons summarize and calculate the neural information originating from all the upstream sensory neurons to generate motor commands and transmit these commands to the downstream motor neurons. Finally, the motor neurons execute the motor commands and carry out evasive movements. Utilizing behavioral experiment and high‐throughput RNA sequencing technology, we have identified the key genes that are changed after aversive learning in the nerve system.^[^ ^32^ ^]^ According to the gene expression patterens in individual neurons and the structural connectionis,^[^ ^33^ ^]^ we detected the neural circuit responsible for the learning of aversive odors of PA14 within the nervous system of *C. elegans*. This neural circuit contains all the neurons that perform the function of learning to avoid the harm caused by the aversive odors of PA14 and the synaptic connections formed by long‐term evolutionary screening among these neurons.

Based on the network topological structure of this neural circuit, we constructed an ANN for image classification using the existing ANN modules. The training results on the public image classification datasets demonstrate that this ANN inspired by the aversive olfactory learning neural circuit in *C. elegans* has higher accuracy, greater consistency and faster convergence rate compared with other traditional ANNs having the same number of parameters. Especially when facing more complex classification tasks, the performance advantages of the ANN constructed inspired by *C. elegans* are more remarkable.

This paper is structured as follows: In Section 2, we describe the detailed behavioral experiment devised to elicit the learning reaction to the aversive olfactory stimuli of PA14. Then, utilizing high‐throughput RNA sequencing technology, we identified the functional neural circuit within the nervous system of *C. elegans* that supports this learned behavior. Section 3 specifies the design of an ANN for image classification, which is inspired and constructed by the network topological structure of the functional neural circuits related to aversive olfactory learning identified in *C. elegans*. In Section 4, we report the results and compare the performance of different ANNs on various public image classification datasets. The paper concludes with a discussion of our findings in Section 5.

## 2 Functional Neural Circuit of Aversive Olfactory Learning in *C. elegans*

**Figure 1:**
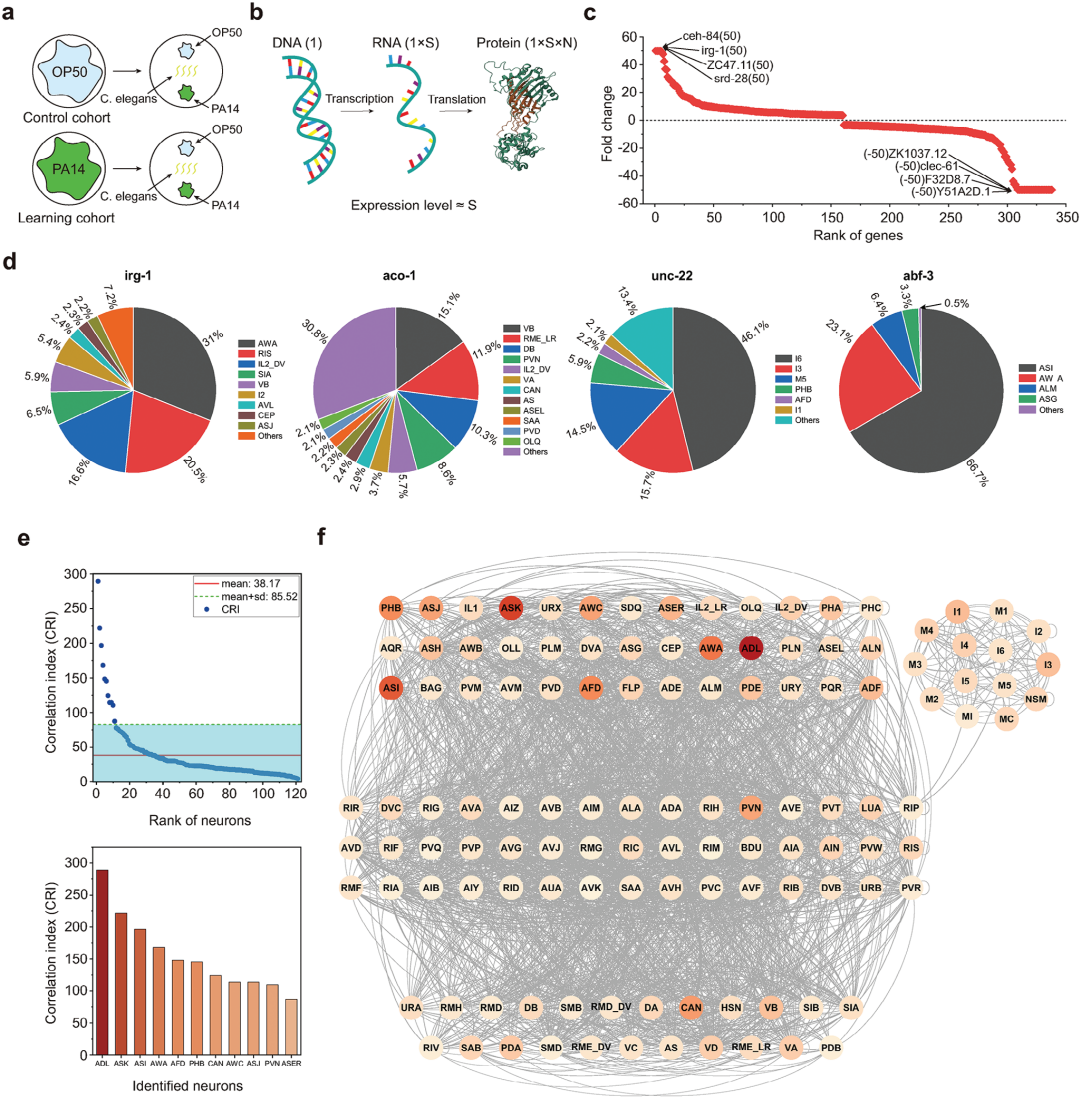
a) Implementation principle of the behavioral experiment for learning to avoid aversive olfactory stimuli in *C. elegans*. b) Basics of high‐throughput RNA sequencing technology. c) Gene expression fold changes between the learning and control cohorts. Positive values indicate upregulation and negative values denote downregulation. To minimize the measurement error, we restrict the fold changes to lie within the range of [–50, 50]. d) Gene expression percentage in specific functional neurons, excluding functional neurons with expression percentage below 2%. e) Identification of functionally correlated neurons associated with aversive olfactory learning in *C. elegans*. f) The functional neural network of *C. elegans*. The width of edges is proportional to the number of synapses *EW* between two functional neurons, while the intensity of color within the functional neurons indicates the magnitude of *CRI*.

The nervous system of *C. elegans* is composed of 302 neurons and over 7000 synaptic connections. To emphasize functional connectivity over network size, we have combined neurons with analogous functions into a single functional unit. For example, the ADAL (ADA Left) and ADAR (ADA Right) neurons have been unified into a single functional ADA neuron, with their synaptic connections aggregated accordingly. In contrast, neurons serving distinct functions, such as ASEL and ASER, have been retained as separate functional entities. Through the process of function clustering, we have effectively simplified *C. elegans*' neural network into a more refined topological structure containing 121 functional neurons (as depicted in **Figure** **1**f).

The following procedural steps outline the behavioral experiment designed to evaluate the learning ability of *C. elegans* in avoiding the aversive olfactory stimuli of PA14 (as depicted in Figure 1a). The experiment start with two identical cohorts of *C. elegans*, both raised under standard laboratory conditions. Each cohort is separately exposed to two kinds of bacterial strains for a duration of 4 h: the non‐pathogenic escherichia coli OP50 and the pathogenic PA14.^[^ ^34^, ^35^, ^36^ ^]^ After exposure, the worms from each cohort are placed at the center of a new culture plate. The new culture plate is uniformly coated with op50 and pa14 at two centrosymmetric points, thereby creating the two‐choice experiment of olfactory stimuli. Finally, the experiment is concluded with a quantification of the preference proportions for both OP50 and PA14 among the worms in each cohort. The results of the experiment show that *C. elegans* can form aversive memories associated with the odors of PA14 after a 4‐h learning period, which is demonstrated by their preferential avoidance of the pathogenic strain.^[^ ^37^, ^38^ ^]^

We aim to elucidate the molecular mechanism of gene expression regulation that underlies aversive olfactory learning in *C. elegans* by employing high‐throughput RNA sequencing technology (as shown in Figure 1b). The core concept of this technology is based on the transcription of a single gene within DNA into *S* RNA molecules. These *S* RNA molecules are then translated into *S* × *N* proteins, where *N* is a fixed constant. The level of a gene expression is thus represented by *S*. We have quantified the genes expression levels in both the control and learning cohorts as illustrated in Figure 1a. Comparative analysis of genes expression between the two cohorts has led to the identification of 338 genes exhibiting differential expression, where positive values signify upregulation and negative values denote downregulation, as depicted in Figure 1c. However, these results did not identify the specific functional neurons responsible for these genes expression variations. Prior studies have established the expression patterns of these 338 genes in individual neurons of naive *C. elegans*. Figure 1d provides a graphical illustration of selected examples. A plausible inference is that the expression of the 338 genes will change in all neurons that express them. The changes will be proportional to their expression in naive *C. elegans* during aversive olfactory learning. To measure the activity of functional neurons in association with aversive olfactory learning, we introduce the correlation index (CRI). The formula for computing the CRI is as follows:

(1) $$CRI_{i}=\sum_{j=1}^{N}\left(W_{ji}\times \left|M_{j}\right|\right).$$

Here, *CRI*_*i*_ represents the correlation index of neuron *i*, *W*_*ji*_ is the expression proportion of gene *j* in neuron *i* within naive *C. elegans*, *M*_*j*_ is the fold change in gene expression of gene *j* and *N* is the total number of genes that showed differential expression after a 4‐h learning period. The *CRIs* for 121 functional neurons were computed independently and the results are summarized in **Table** **1**. Employing statistical analysis on the *CRIs* of all the functional neurons listed in Table 1, we have identified 11 functional neurons that display a significant correlation to aversive olfactory learning in *C. elegans*, as presented in Figure 1e. Interestingly, majority of the 11 identified learning‐related functional neurons are sensory neurons, while the rest are one interneuron PVN and one motor neuron CAN.

**Table 1:** Correlation indexes of functional neurons.

Neuron	*CRI*	Neuron	*CRI*	Neuron	*CRI*	Neuron	*CRI*
I1	76.20	SDQ	15.04	RIS	38.09	RID	11.17
I2	24.14	AQR	18.17	ALA	21.37	AVB	11.00
I3	73.24	PQR	21.58	PVQ	6.22	AVA	33.75
I4	48.71	ALM	16.92	ADA	13.50	PVC	12.56
I5	33.67	AVM	15.57	RIF	18.14	RIP	11.22
I6	19.02	PVM	21.70	BDU	16.73	URA	29.45
M1	24.11	PLM	16.86	PVR	10.77	RME_LR	30.14
M2	27.29	FLP	45.12	AVF	12.41	RME_DV	17.44
M3	23.28	DVA	22.71	AVH	22.14	RMD_DV	17.48
M4	46.37	PVD	23.26	PVP	17.54	RMD	13.30
M5	20.83	ADE	23.08	LUA	47.80	RIV	8.79
MC	39.46	PDE	66.95	PVN	110.22	RMH	16.11
MI	11.88	PHA	64.58	AVG	19.56	SAB	42.23
NSM	40.44	PHB	145.40	DVB	20.38	SMD	9.65
ASI	196.67	PHC	15.94	RIB	26.92	SMB	12.98
ASJ	114.12	IL2_DV	51.08	RIG	12.16	SIB	17.72
AWA	168.32	IL2_LR	35.32	RMG	7.73	SIA	25.58
ASG	39.42	CEP	16.85	AIB	5.76	DA	37.78
AWB	44.72	URY	22.48	RIC	31.01	PDA	59.73
ASEL	29.98	OLL	10.73	SAA	15.27	DB	34.03
ASER	87.19	OLQ	19.05	AVK	4.13	AS	19.04
ADF	77.80	IL1	32.00	DVC	36.16	PDB	8.43
AFD	148.39	AIN	42.26	AVJ	11.69	VA	54.20
AWC	114.14	AIM	12.75	PVT	30.11	VB	71.87
ASK	221.88	RIH	22.59	AVD	15.52	VD	52.25
ASH	70.08	URB	22.63	AVL	12.18	CAN	124.36
ADL	289.51	RIR	18.92	PVW	29.92	HSN	27.93
BAG	17.44	AIY	10.83	RIA	8.25	VC	20.47
URX	27.61	AIA	22.60	RIM	5.32		
ALN	47.57	AUA	15.91	AVE	9.81		
PLN	29.92	AIZ	9.92	RMF	18.69		

The neural information flow within the brain of *C. elegans* originates from sensory neurons, then traverses through interneurons and finally culminates at motor neurons. Following the sequence of neural information flow, we have configured the neural network of *C. elegans*, as depicted in Figure 1f. In this network representation, sensory neurons are located in the upper layer, with interneurons in the middle layer and motor neurons in the lower layer. The intensity of the color within each functional neuron correlates with the magnitude of the CRI, offering a visual indication of their respective activities in the context of aversive olfactory learning. The width of the edge from functional neuron *i* to functional neuron *j* *EW*_*ij*_ is determined by the total number of chemical and electrical synapses contained in this synaptic connection and it reflects the strength of the communication ability. *EW*_*ij*_ is quantified by the following equation:

(2) $$EW_{ij}=C_{ij}+E_{ij}.$$

*EW*_*ij*_ represents the weighted connection strength from functional neuron *i* to *j*. *C*_*ij*_ and *E*_*ij*_ respectively denote the number of chemical and electrical synapses contained in this synaptic connection. It is important to note that chemical synapses facilitate unidirectional transmission of neural information, which is often expressed as *C*_*ij*_ ≠ *C*_*ji*_. On the other hand, electrical synapses permit bidirectional communication, signified by *E*_*ij*_ = *E*_*ji*_.

By utilizing the connectivity map of functional neurons as shown in Figure 1f and in combination with the 11 functional neurons having a strong correlation with aversive olfactory learning as shown in Figure 1e, we have successfully identified the neural circuit responsible for aversive olfactory learning within the neural network of *C. elegans*. Two criteria are used for the neural circuit: 1. the whole neural circuit contains three levels: sensory neurons, interneuron neurons and motor neurons; 2. the neural circuit only contain the strong connected neurons ignoring the neurons with weak synaptic connections. Because identified 11 functional neurons are devided into three types according to their functional roles, the detailed identification procedure is presented in the following steps with three scenarios:

**Scenario One: Sensory Neuron Initiation**

**Figure 2:**
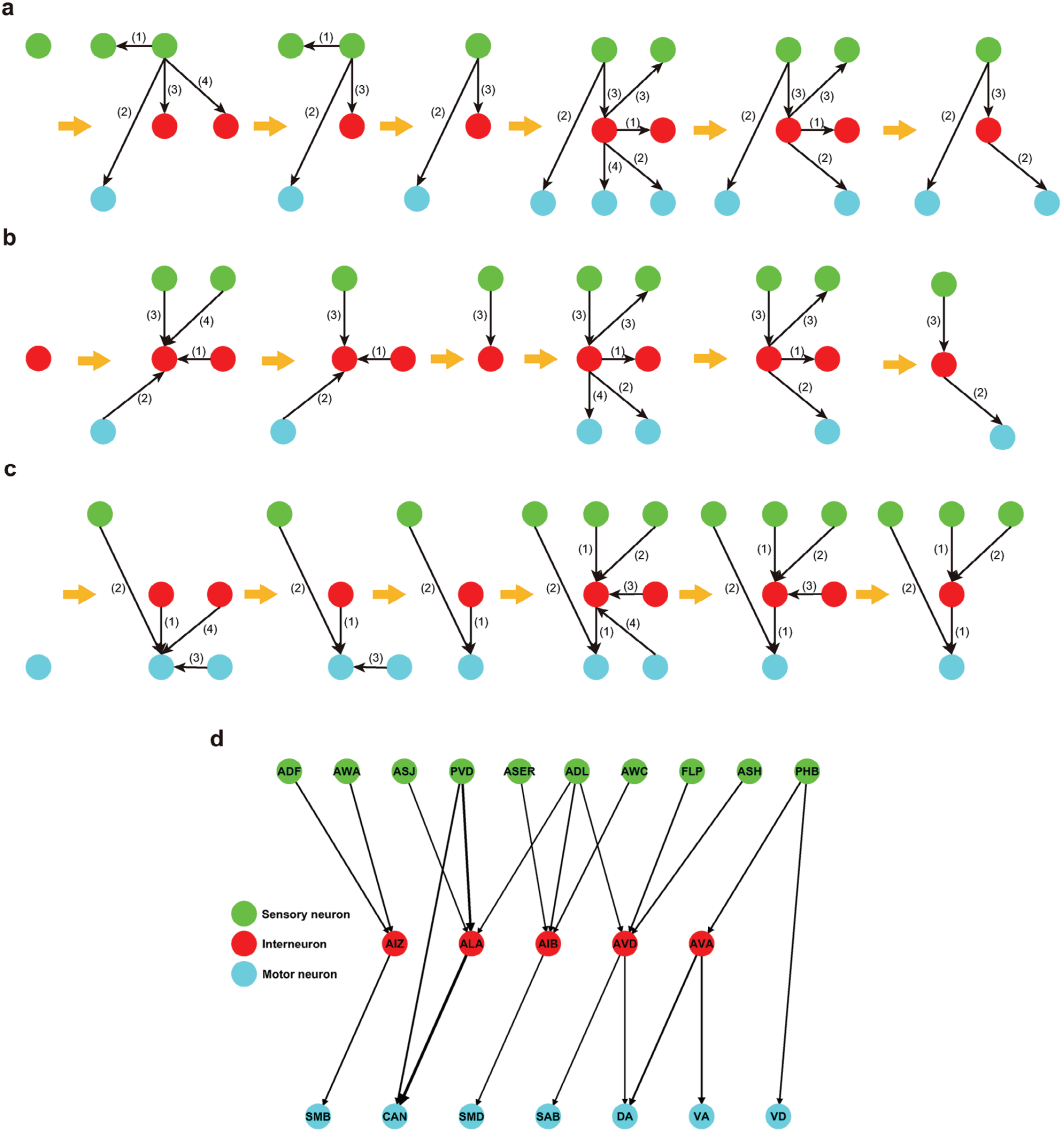
a) The steps of extending the functional neural circuit with sensory neuron as starting neuron. The bracketed value next to arrow line represents the rank determined by the number of synapses *EW* contained in this synaptic connection from large to small. b) The steps of extending the functional neural circuit with interneuron as intermediate neuron. c) The steps of extending the functional neural circuit with motor neuron as terminal neuron. d) The functional neural circuit associated with aversive olfactory learning of *C. elegans*, which consist of 22 functional neurons (10 sensory neurons, 5 interneurons. and 7 motor neurons) and 21 synaptic connections.

This scenario involves using identified nine sensory neurons as the starting points to trace two steps of downstream neurons for discovering the functional neural circuits. The information flow from individual neuron is possitive correlated with synaptic connection number with that neuron. Therefore, The three strongest presynaptic connections of each starting neuron are oriented toward second‐step neurons. If second‐step neurons comprise interneurons or motor neurons, the interneurons or motor neurons and the strong synapses from starting neurons to them will be retained. Then, the strong presynapses of each interneuron in second‐step neurons are oriented toward third‐step neurons. If third‐step neurons comprise motor neurons, the motor neurons and the strong synapses from interneurons in second‐step to them will be retained (**Figure** **2**a).

**Scenario Two: Interneuron PVN as Intermediary**

This scenario involves using identified one interneuron PVN as the intermediate neuron of the functional neural circuits to trace the upstream neurons and downstream neurons. The three strongest postsynaptic connections of the interneuron are oriented toward first‐step neurons. If first‐step neurons comprise sensory neurons, the sensory neurons and the strong synapses from them to the interneuron will be retained. The three strongest presynaptic connections of the interneuron are oriented toward third‐step neurons. If third‐step neurons comprise motor neurons, the motor neurons and the strong synapses from the interneuron to them will be retained (Figure 2b).

**Scenario Three: Motor Neuron CAN as Terminal**

The third scenario involves using identified one motor neuron CAN as the terminal neuron of the functional neural circuits to trace two steps of upstream neurons. The three strongest postsynaptic connections of the motor neuron are oriented toward second‐step neurons. If second‐step neurons comprise sensory neurons or interneurons, the sensory neurons or interneurons and the strong synapses from them to the motor neuron will be retained. The three strongest postsynaptic connections of each interneuron in second‐step neurons are oriented toward first‐step neurons. If first‐step neurons comprise sensory neurons, the sensory neurons and the strong synapses from them to interneurons in second‐step neurons will be retained (Figure 2c).

Finally, the unretained neurons and synapses are eliminated to obtain the functional neural circuit responsible for aversive olfactory learning of *C. elegans*, which comprises 22 functional neurons (10 sensory neurons, 5 interneurons, and 7 motor neurons) and 21 synaptic connections (Figure 2d). In comparison with a fully connected neural network having the same number of neurons, the sparseness of the functional neural circuits is 4%.

## 3 Artificial Neural Networks for Image Classification

Image classification stands as a central task within the field of computer vision, aiming to differentiate between various categories of images based on their distinctive features. Mathematically speaking, it involves the discovery of a function capable of mapping the pixel intensities of an image to a specific class label. Humans, being endowed with extensive prior knowledge, can categorize images effortlessly and subconsciously. In contrast, for computers, discerning image categories solely from pixel values is a nontrivial challenge. Prior to the deep learning revolution, image classification often depended on machine learning models that employed manually crafted features. This process was highly dependent on expertise and iterative experimentation. The emergence of deep learning has significantly alleviated the requirement for manual feature engineering. Artificial neural networks, especially those with a large number of parameters, are now capable of extracting features and classifying images autonomously. Currently, most of the models boasting excellent image classification performance are convolutional ANNs. Unlike traditional fully connected neural networks, CNNs utilize convolution operations to capture local features in the input images. By stacking multiple ANN modules and refining their interaction, the accuracy of image classification can be significantly improved.

**Figure 3:**
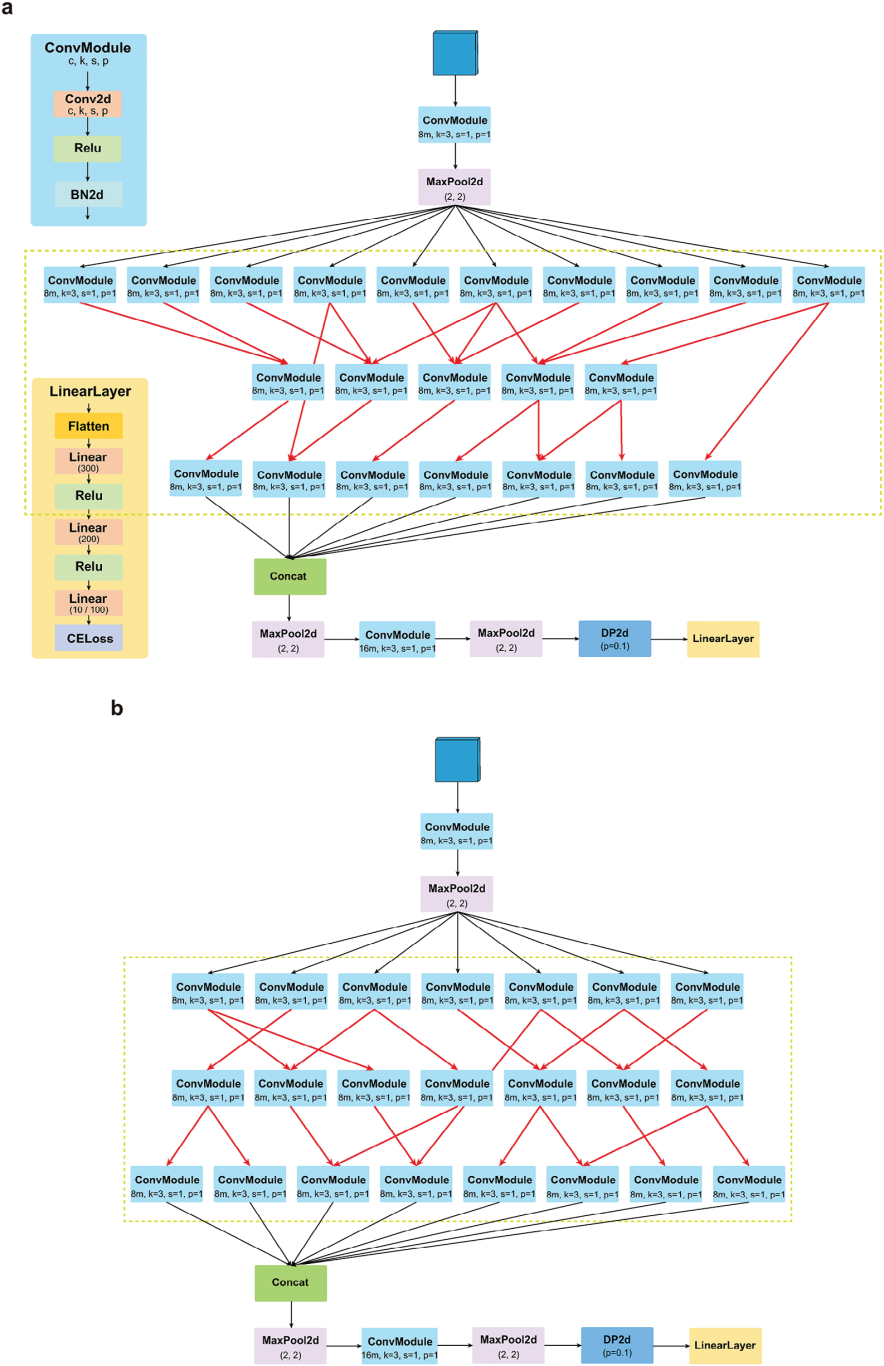
a) The framework map of the artificial neural network for image classification is inspired by the neural circuit that are responsible for aversive olfactory learning in *C. elegans*. The part framed by the green dotted line is the functional neural circuit module of *C. elegans*. b) The framework map of the artificial neural network for image classification is inspired by the randomized neural circuit. The part framed by the green dotted line is the randomized functional neural circuit module.

Determining the optimal combination architecture of ANN modules to build an ANN for image classification is a challenging task. The functional neural circuit responsible for aversive olfactory learning of *C. elegans* have undergone long‐term evolutionary screening, exhibiting a compact structure and high computing speed. Therefore, taking inspiration from the topological structure of the functional neural circuit depicted in Figure 2d, we have designed an ANN based on convolution layers, pooling layers and fully connected layers for image classification, as shown in **Figure** **3**a. In this ANN, we have innovatively introduced the neural circuit module that is framed by the green dotted line. This neural circuit module transforms the neurons within the functional neural circuit, which is responsible for the aversive olfactory learning of *C. elegans*, into convolutional layers and converts the synaptic connections between neurons into the information flow paths among convolutional layers. Thus, this ANN has a computing mode similar to that of the brain of *C. elegans*. To emphasize the advantages of the network design illustrated in Figure 3a, we additionally constructed a comparative ANN as shown in Figure 3b. This comparative ANN features a randomized module of neural circuit. Notably, this randomization is carried out while maintaining an equal number of functional neurons and synaptic connections as those in the original network.

The topological structures of ANNs in Figure 3 are intricate and significantly different from the sequential structures typically found in traditional ANNs for image classification. The pixel information of the images initially undergoes a convolution operation for initial feature extraction. Subsequently, the feature maps are fed into the neural circuit module for computing in the brain‐like mode of *C. elegans*. The intricate connections within the neural circuit module enable a more nuanced and powerful representation of the feature maps. Finally, the feature maps output by the neural circuit module undergo a convolution operation and a series of fully connected mappings to obtain the image classification results. The pooling operation is employed to reduce the size of the feature maps. In addition, we have set a variable m to scale up or down the number of parameters within the ANNs. By assigning different values to variable m, we can generate image classification models that possess different quantities of parameters. It is widely acknowledged that models with a larger quantity of parameters generally demonstrate better performance. However, they also require more computing resources. The balance between the quantity of parameters and the computing resources is a crucial factor in attaining optimal performance for a model. An excessive number of parameters can also lead the model to the performance nadir of overfitting.

## 4 Results

All the results were obtained on the Windows11 platform (Intel i7‐12700KF CPU, Nvidia RTX4090 GPU) within the software environment (Python3.9, CUDA11.8, PyTorch2.3). We performed result tests on multiple public datasets for image classification. The details of the datasets are listed in **Table** **2**. We also introduced additional ANNs with excellent performance in image classification for more extensive performance comparisons, such as LeNet‐5,^[^ ^1^ ^]^ AlexNet,^[^ ^39^ ^]^ VGG‐16^[^ ^40^ ^]^ and GoogLeNet^[^ ^2^ ^]^ (as shown in Figure A1, Supporting Information). For these ANNs, we also set a variable m to scale the number of parameters for generating different image classification models. The corresponding relationship between the number of parameters and the variable m of all image classification models when tested on the CIFAR10 dataset is presented in **Table** **3**. The tests were designed to evaluate three aspects of performance: 1) The overall classification accuracy within all represented categories. 2) The consistency of classification accuracy among different categories. 3) The rate of convergence of classification loss, which is indicative of the model's learning efficiency.

**Table 2:** The public datasets for image classification.

Dataset	Color channel	Size	Category	Training set	Test set
MNIST	1	28 × 28	10	60000	10000
FashionMNIST	1	28 × 28	10	60000	10000
CIFAR10	3	32 × 32	10	50000	10000
CIFAR100	3	32 × 32	100	50000	10000

**Table 3:** The quantities of parameters of models (Based on CIFAR10).

Worm		Randomized		LeNet‐5		AlexNet		VGG‐16		GoogLeNet	
m	parameters	m	parameters	m	parameters	m	parameters	m	parameters	m	parameters
1	151142	1	150558	1.5	138364	0.10	142273	0.035	157407	0.15	131765
2	262814	2	260494	2.0	244962	0.13	243934	0.045	272787	0.20	235004
3	397526	3	392318	2.5	381800	0.16	371351	0.055	406275	0.25	379046
4	555278	4	546030	3.0	548878	0.19	528107	0.065	564983	0.30	532904
5	736070	5	721630	3.5	746196	0.22	709334	0.075	750708	0.35	725519
6	939902	6	919118	4.0	973754	0.25	920634	0.085	961747	0.40	947089
7	1166774	7	1138494	4.5	1231552	0.28	1132149	0.095	1200761	0.45	1198960
8	1416686	8	1379758	5.0	1519590	0.31	1391399	0.105	1463940	0.50	1502690

**Figure 4:**
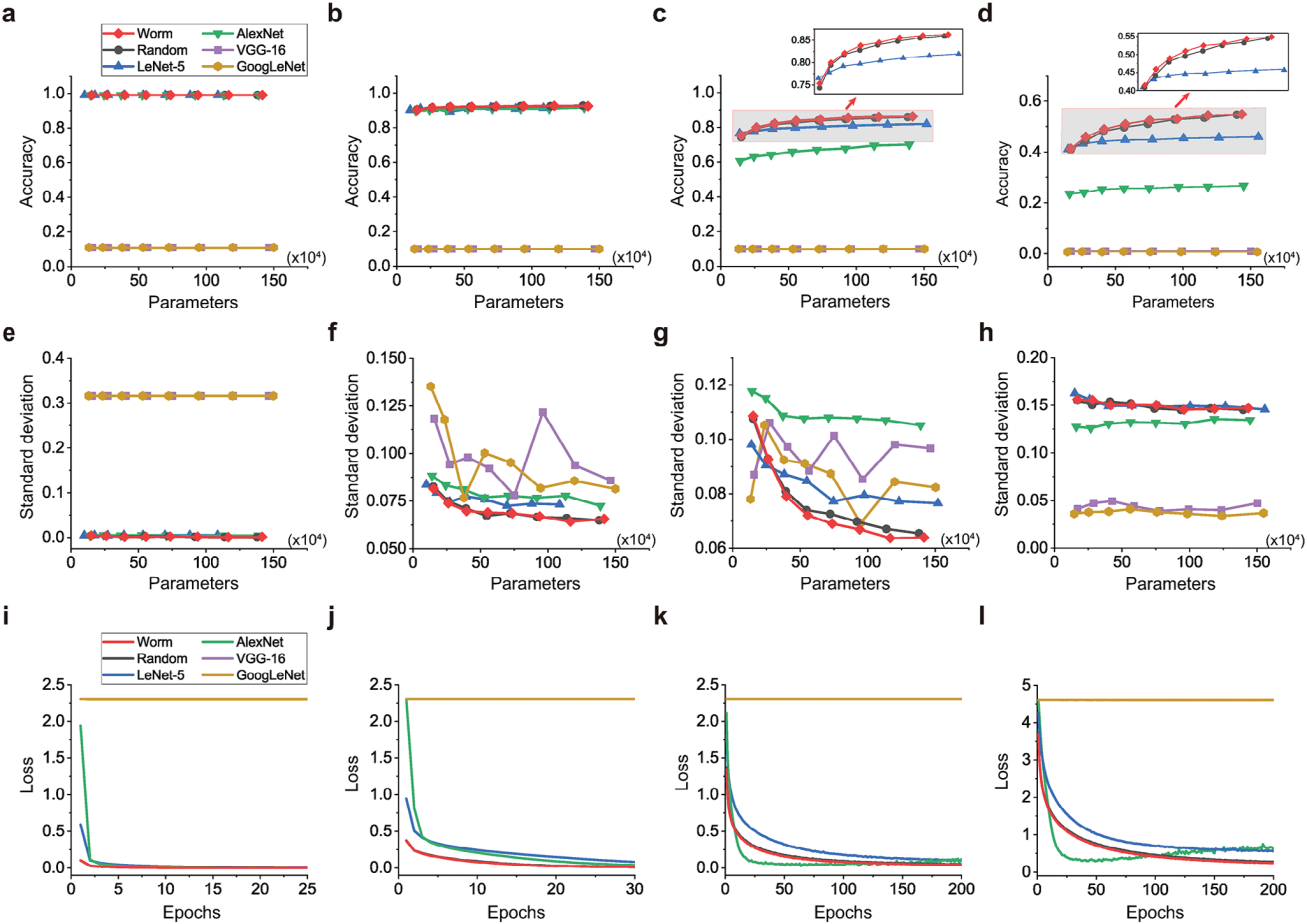
The accuracy of classification across all categories on MNIST (a), FashionMNIST (b), CIFAR10 (c), CIFAR100 (d). The consistency of image classification accuracy among different categories on MNIST (e), FashionMNIST (f), CIFAR10 (g), CIFAR100 (h). The rate of convergence of classification loss on MNIST (i), FashionMNIST (j), CIFAR10 (k), CIFAR100 (l).

**Figure** **4**a–d presents the classification accuracy of models with different parameters on four public datasets. On the simple datasets, MNIST and FashionMNIST, worm models inspired by the aversive olfactory learning neural circuit in *C. elegans*, random models inspired by the randomized neural circuit, LeNet and AlexNet all possess high classification accuracy when equipped with different parameters. On the complex datasets, CIFAR10 and CIFAR100, worm models and random models show obvious classification accuracy advantages over LeNet and AlexNet under each parameters size. However, models of VGG‐16 and GoogLeNet at all listed parameter sizes cannot achieve image classification task on the four public datasets. The reason is that the number of parameters in the original models of VGG‐16 and GoogLeNet is extremely large, 13430 × 10^4^ and 598 × 10^4^. When we significantly scale down the number of parameters, the functionality of the models is damaged. Figure 4e–g indicates that worm models and random models still have the best consistency of image classification accuracy among different categories. In Figure 4h, the classification accuracy of AlexNet, VGG‐16 and GoogLeNet is extremely low in all categories, which makes the target of consistency meaningless. Furthermore, we analyze the convergence rate of the classification loss of the models, each of which has the listed maximum parameters among each type of ANNs. On the MNIST and FashionMNIST datasets, the worm and random models have the fastest convergence speeds. On the CIFAR10 and CIFAR100 datasets, AlexNet model has the fastest convergence speed. A special phenomenon is that the classification loss drops rapidly at first and then rises slowly, which indicates that the model of AlexNet has overfitted. Upon comprehensive comparison of all the models, worm models achieve the best performance, with random models ranking second. The performance advantage is more obvious when the models are tasked with more complex image classification challenges.

## 5 Conclusion and Discussion

This study explores the potential of bio‐inspired design in the development of ANNs for image classification. Through a behavioral experiment and high‐throughput RNA sequencing technology, as well as the connectome data, this study identified the neural circuit responsible for the learning of aversive odors of PA14 within the nervous system of *C. elegans*. Based on the network topological structure of this neural circuit, we constructed an ANN for image classification using the existing ANN modules. The test results on the four public image classification datasets show that the ANN inspired by the aversive olfactory learning neural circuit in *C. elegans* has higher accuracy, greater consistency and faster convergence rate compared with other traditional ANNs having the same number of parameters. Particularly when dealing with more complex classification tasks, the performance advantages of the ANN constructed under the inspiration of *C. elegans* become even more remarkable. This suggests that the topological structure of the neural circuits formed by long‐term evolutionary screening of *C. elegans* enhances the ANN's ability to generalize and learn from images, thereby leading to improved performance.

This study provides evidence that bio‐inspired design can remarkably enhance the capabilities of ANNs for image classification, but there are still two limitations. First, we believe that the reason why our classification ANNs achieve performance advantages is that they draw on the simple and efficient neural information computing architecture related to learning to avoid the harm caused by the aversive odors of PA14, which is obtained by *C. elegans* through long‐term evolutionary screening. Nevertheless, since it is impossible to exhaust all potential topological structures of neural circuits, we cannot prove that this neural circuit responsible for the learning of aversive odors of PA14 within the nervous system of *C. elegans* is optimal. Second, since we did not design new ANN modules but only assembled existing convolution layers, pooling layers and fully connected layers according to the functional neural circuit of *C. elegans*, we cannot confirm whether these existing ANN modules are the most appropriate for this neural information computing architecture.

This study opens up new avenues for ANN design and encourages further exploration of biological systems for inspiration in developing more efficient and effective artificial intelligence systems.

## Conflict of Interest

The authors declare no conflict of interests.

## Supporting information

Supporting Information

## Data Availability

Data sharing is not applicable to this article as no new data were created or analyzed in this study.
