# Supplementary material for: An Artificial Neural Network for Image Classification Inspired by the Aversive Olfactory Learning Neural Circuit in Caenorhabditis elegans
Source: Adv Sci (Weinh). 2024 Dec 16;12(7):2410637. doi: 10.1002/advs.202410637 (PMC11831476; doi:10.1002/advs.202410637)
Supplement: Supplementary file 1 — Supporting Information [file ADVS-12-2410637-s001.pdf]

ADVANCED SCIENCE
Open Access

Supporting Information

for *Adv. Sci.*, DOI 10.1002/advs.202410637

An Artificial Neural Network for Image Classification Inspired by the Aversive Olfactory Learning Neural Circuit in *Caenorhabditis elegans*

Xuebin Wang, Chunxiuzi Liu, Meng Zhao, Ke Zhang, Zengru Di and He Liu**

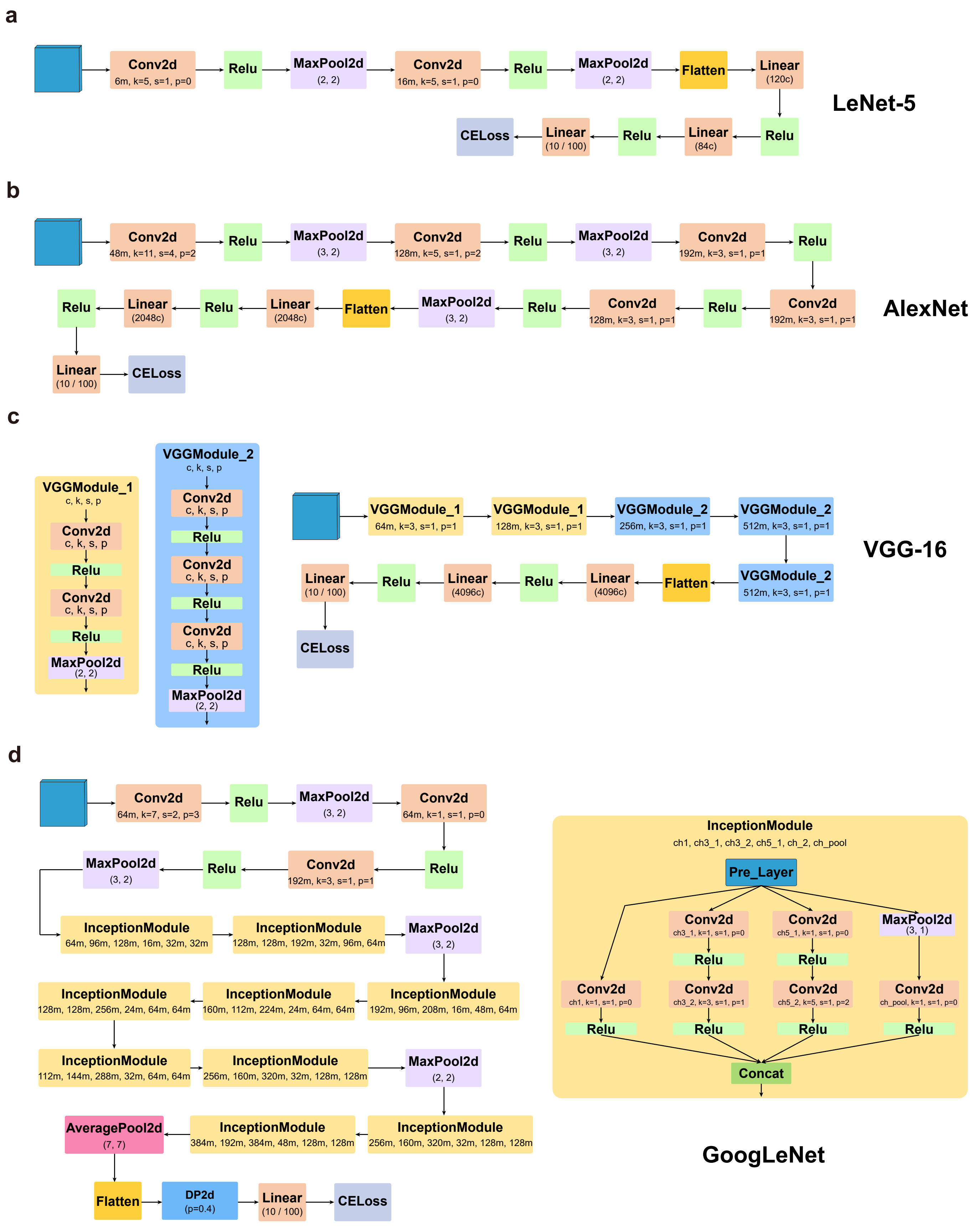

Figure A1.

The framework maps of traditional ANNs with excellent performance for image classification. a) LeNet-5, b) AlexNet, c) VGG-16 and d) GoogLeNet.
